# Correction: Extracellular vesicles from diverse fungal pathogens induce species-specific and endocytosis-dependent immunomodulation

**DOI:** 10.1371/journal.ppat.1014029

**Published:** 2026-03-09

**Authors:** Geneva N. Kwaku, Kirstine Nolling Jensen, Patricia Simaku, Daniel J. Floyd, Joseph W. Saelens, Christopher M. Reardon, Rebecca A. Ward, Kyle J. Basham, Olivia W. Hepworth, Tammy D. Vyas, Daniel Zamith-Miranda, Joshua D. Nosanchuk, Jatin M. Vyas, Hannah Brown Harding

S1 File is omitted from the list of Supporting Information. It can be viewed below.

## Supporting information

S1 File Datapoints. (XLSX)
