# Supplementary figures and images for: Extracellular vesicles from diverse fungal pathogens induce species-specific and endocytosis-dependent immunomodulation
Source: PLoS Pathog. 2025 May 30;21(5):e1012879. doi: 10.1371/journal.ppat.1012879 (PMC12157816; doi:10.1371/journal.ppat.1012879)

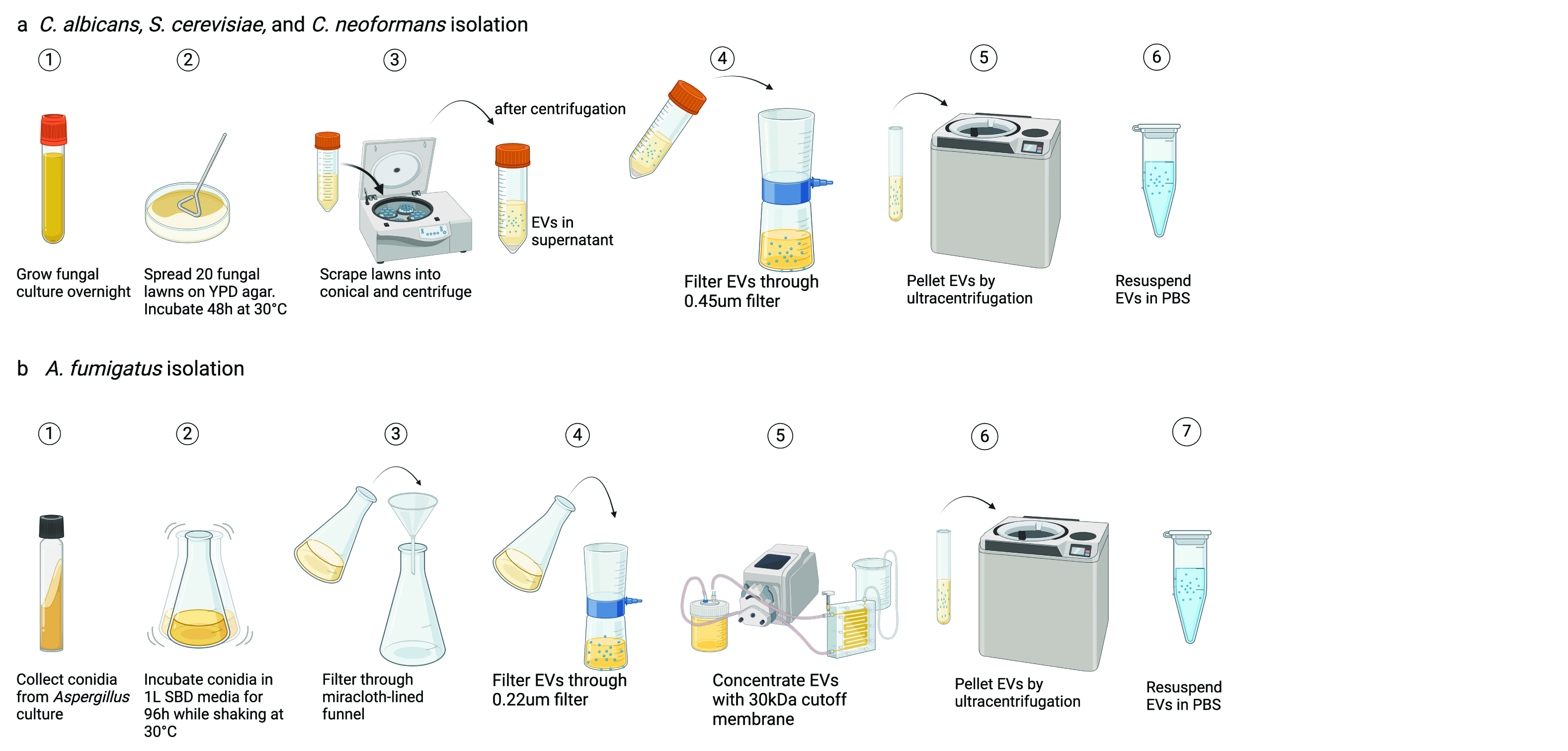

Supplement: S1 Fig — A, Isolation procedures of EVs from C. albicans, S. cerevisiae, and C. neoformans. B, Isolation procedures of EVs from A. fumigatus cultures. Created in BioRender. Brown, H. (2025) https://BioRender.com/g11i747. (TIF) [file ppat.1012879.s001.tif]

a

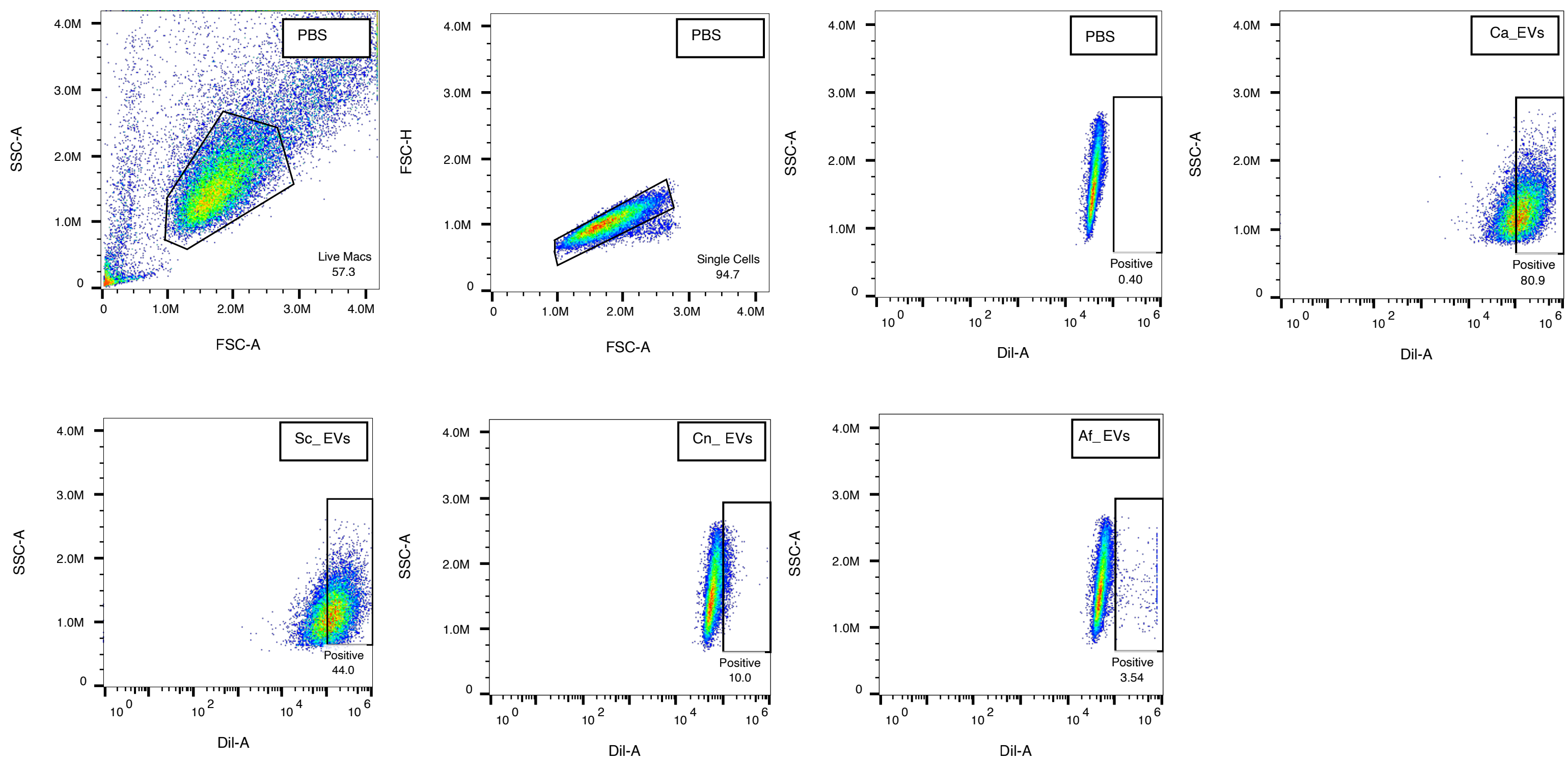

b

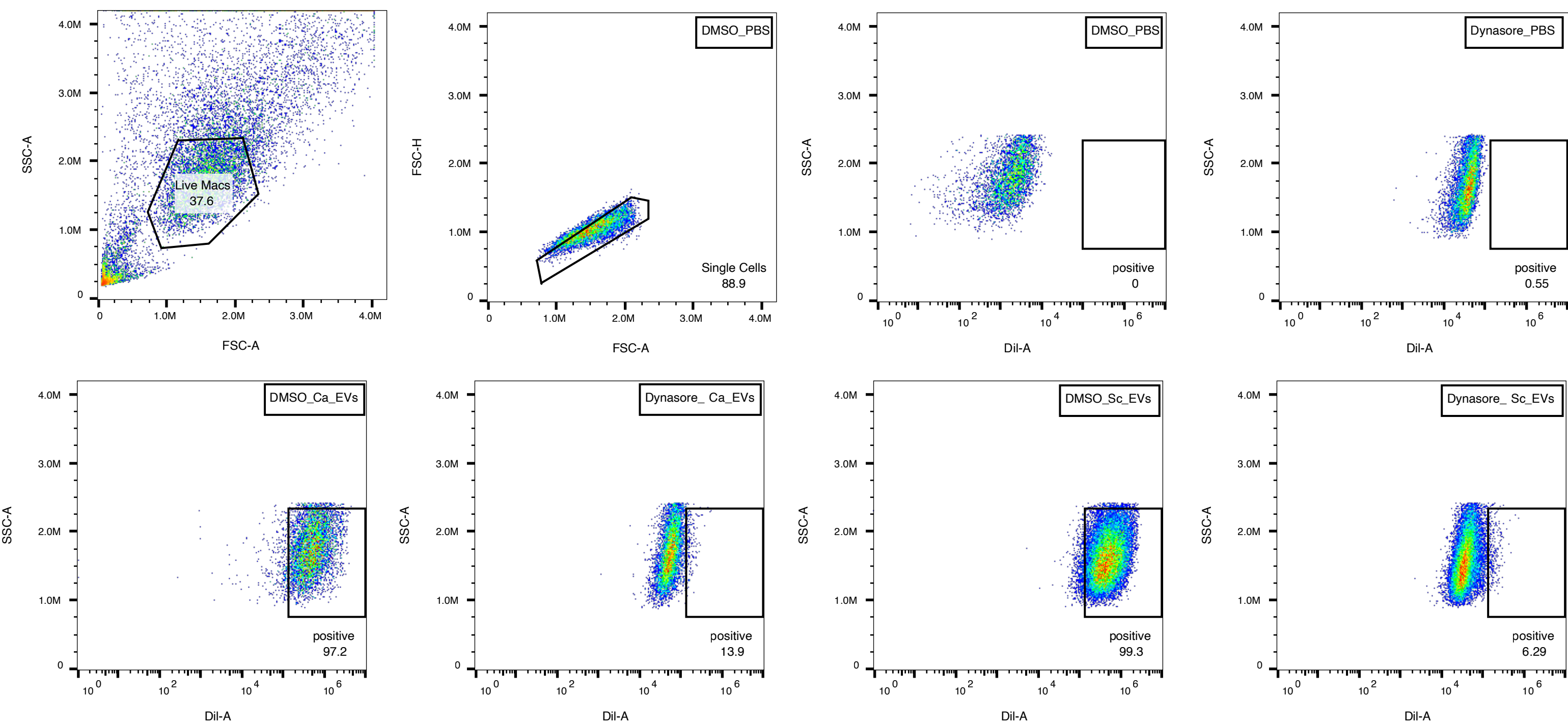

c

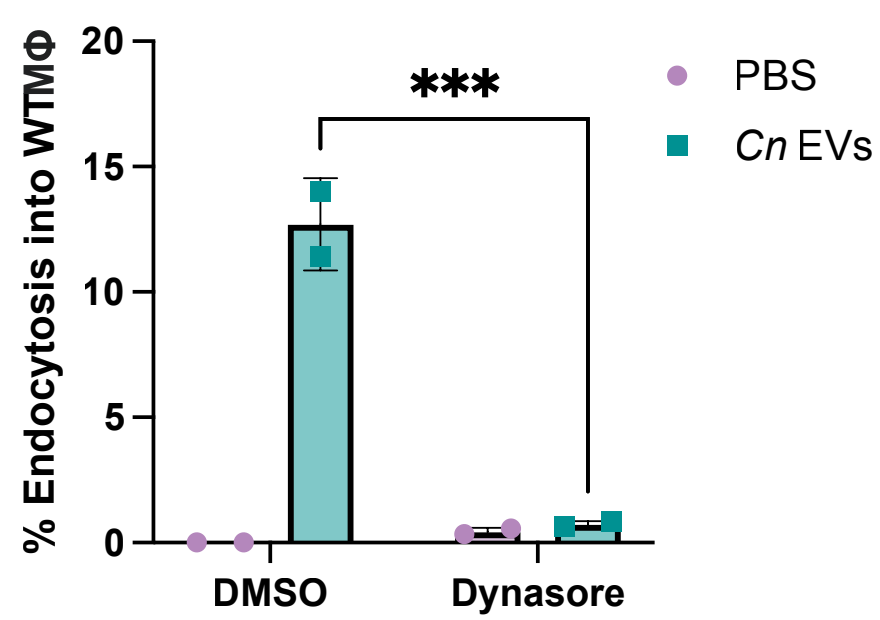

d

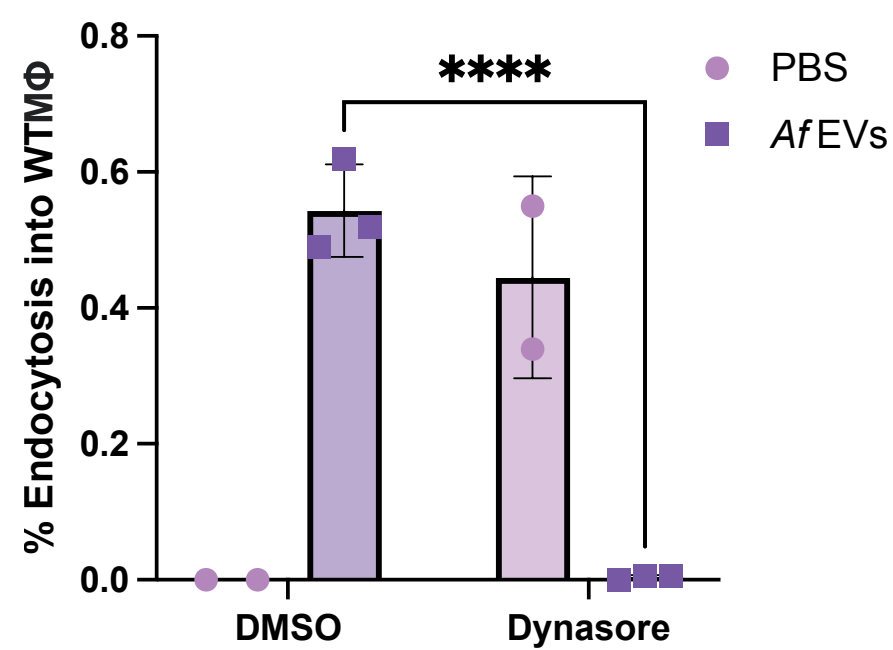

Supplement: S2 Fig — A, Flow cytometry gating strategy for internalization of DiI-labeled fungal EVs by WT macrophages. B, Flow cytometry gating strategy for endocytosis of DiI-labeled fungal EVs by WT macrophages treated with DMSO or 100µM Dynasore. C, Percent endocytosis of Cn EVs with murine macrophages treated with DMSO or 100µM dynasore. Significance assessed by a two-way ANOVA and uncorrected Fisher’s LSD test, ***p = 0.0002 vs respective DMSO controls, n = 2. (5x10^10^ EVs/mL added per stimulation). D, Percent endocytosis of Af EVs with murine macrophages treated with DMSO or 100µM dynasore. Significance assessed by a two-way ANOVA and uncorrected Fisher’s LSD test, ****p=<0.0001 vs respective DMSO controls, n = 3. (5x10^10^ EVs/mL added per stimulation). (PDF) [file ppat.1012879.s002.pdf]

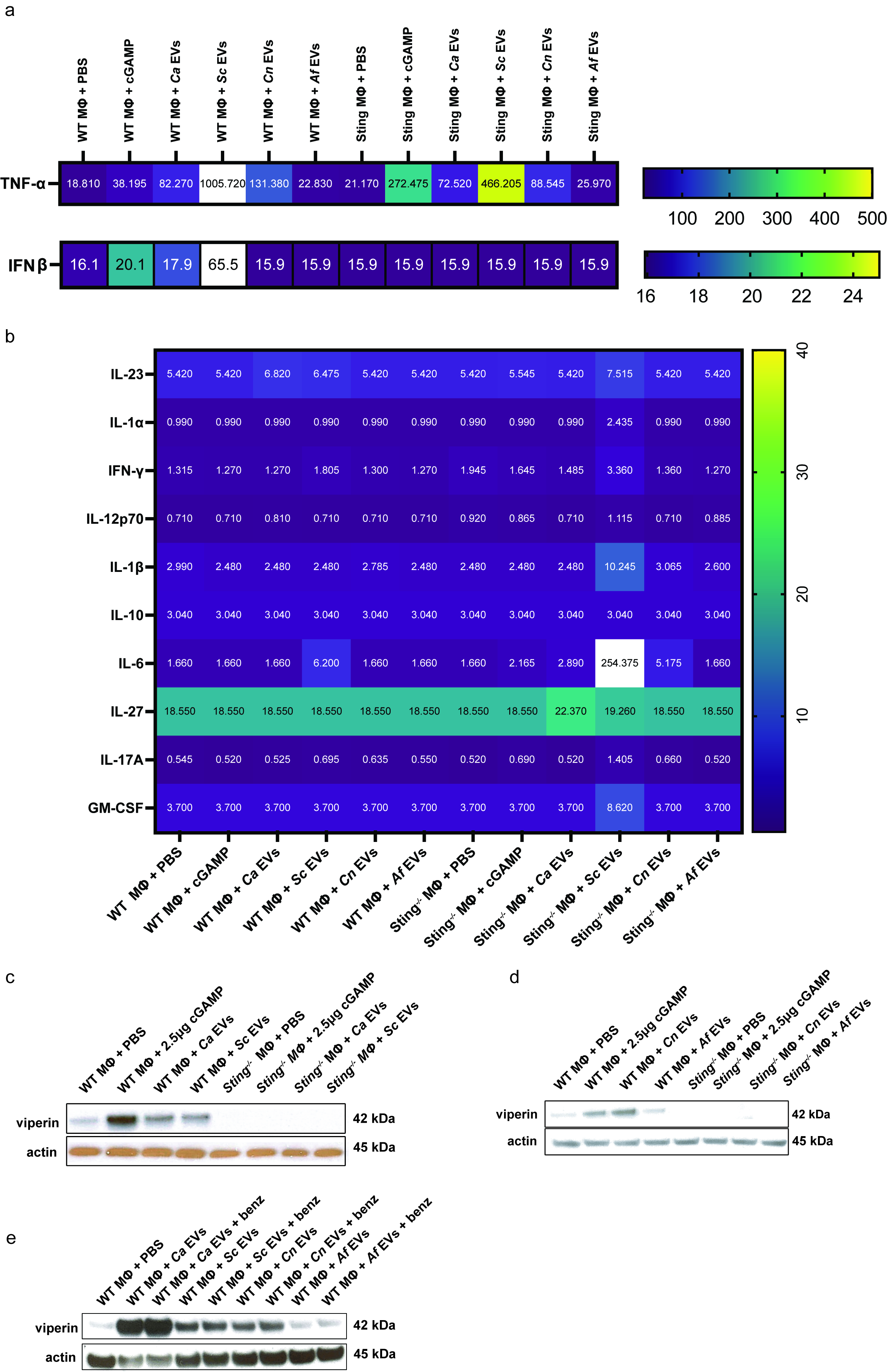

Supplement: S3 Fig — A, Heat maps of TNFα and IFNβ secreted by WT and Sting-/- macrophages when stimulated by PBS, cGAMP, Ca EVs, Sc EVs, Cn EVs, and Af EVs (EVs added at 1x10^10^ EVs/mL). B, Heat map of 10 additional cytokines secreted by WT and Sting-/- macrophages when stimulated by PBS, cGAMP, Ca EVs, Sc EVs, Cn EVs, and Af EVs (EVs added at 1x10^10^ EVs/mL). C, Immunoblots of viperin and actin in WT and Sting-/- macrophages stimulated by PBS, 2.5µg cGAMP, Ca EVs, or Sc EVs (EVs added at 1x10^10^ EVs/mL). D, Immunoblot of viperin and actin in WT and Sting-/- macrophages stimulated by PBS, 2.5µg cGAMP, Cn EVs, or Af EVs (EVs added at 1x10^10^ EVs/mL). E, Immunoblot of viperin and actin in WT macrophages stimulated by PBS, benzonase-treated EVs, and untreated EVs (EVs added at 5x10^10^ EVs/mL). (TIF) [file ppat.1012879.s003.tif]

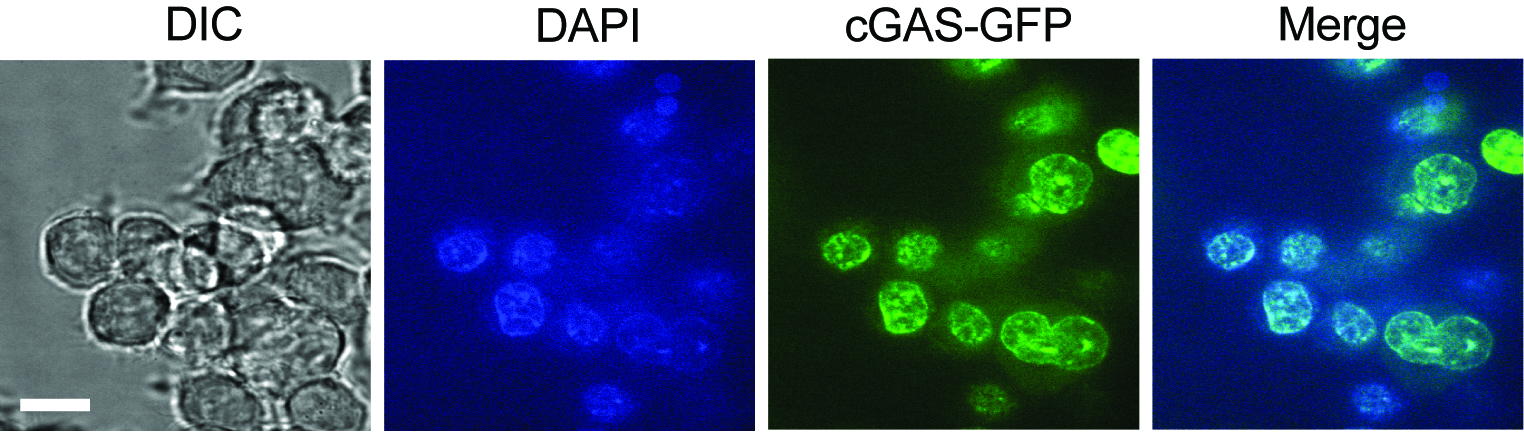

Supplement: S4 Fig — A, Localization of the nuclei (panel 2, blue) and cGAS (panel 3, green) of cGAS-GFP expressing macrophages in an unstimulated state. Macrophages were stained with DAPI for visualization of the nucleus. (TIF) [file ppat.1012879.s004.tif]

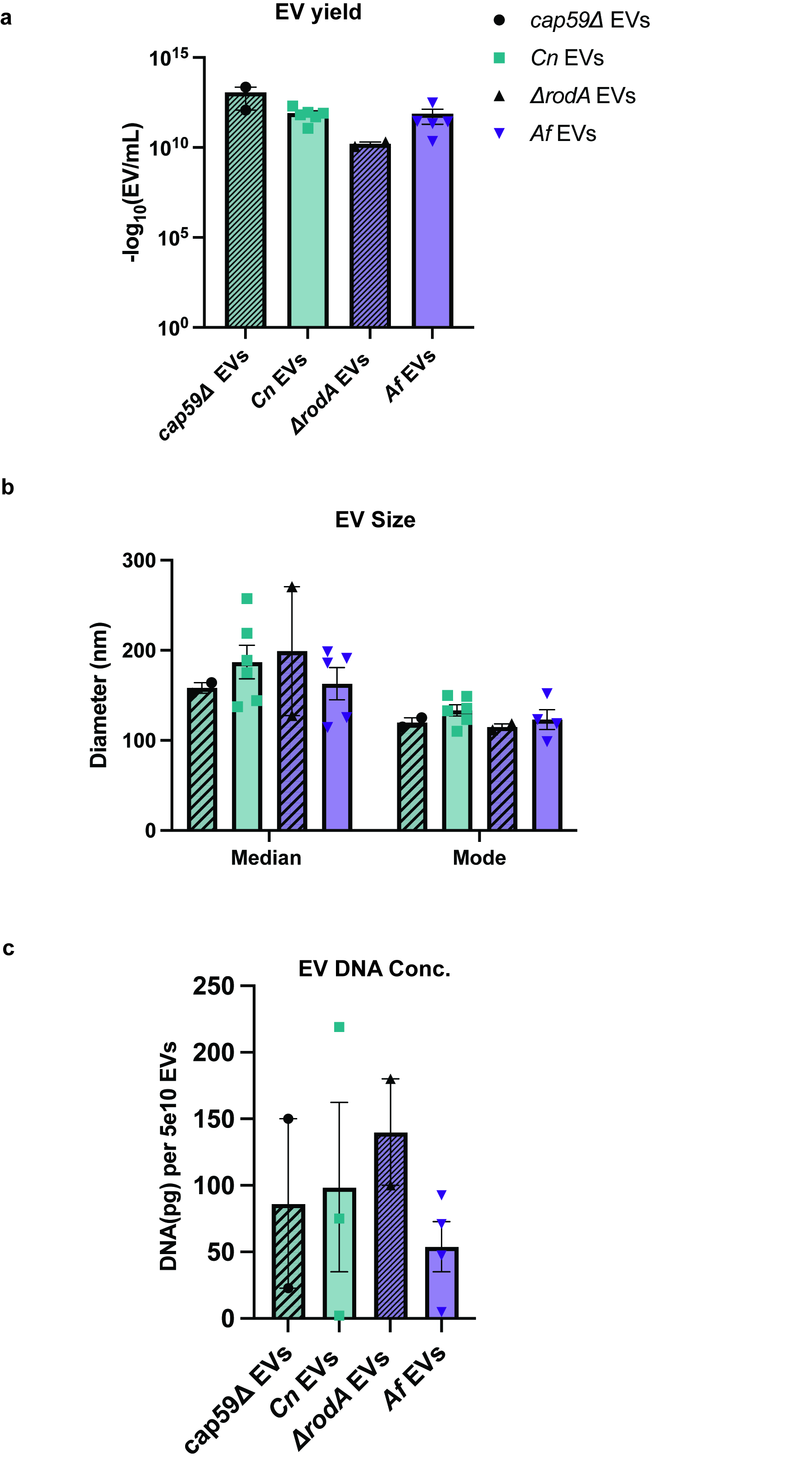

Supplement: S5 Fig — A, The -log10 values of EV concentration from standard isolation preps of cap59∆ EVs and ΔrodA EVs compared to Cn EVs and Af EVs. Significance assessed using an ordinary one-way ANOVA and Tukey’s multiple comparisons test with no significance determined, n ≥ 2. B, The median and mode diameters of cap59∆ EVs and ΔrodA EVs. Significance assessed using an ordinary one-way ANOVA and Tukey’s multiple comparisons test with no significance determined, n ≥ 2. C, The average DNA concentration (pg per 5x10^10^ EVs) of cap59∆ EVs and ΔrodA EVs compared to Cn EVs and Af EVs. Significance assessed using an ordinary one-way ANOVA and Tukey’s multiple comparisons test with no significance determined, n ≥ 3. (TIF) [file ppat.1012879.s005.tif]
